# Finding Possible Weakness in the Runoff Simulation Experiments to Assess Rill Erosion Changes without Non-Intermittent Surveying Capabilities

**DOI:** 10.3390/s20216254

**Published:** 2020-11-02

**Authors:** Alexander André Remke, Jesus Rodrigo-Comino, Stefan Wirtz, Johannes B. Ries

**Affiliations:** 1 Department of Physical Geography, University of Trier, 54286 Trier, Germany; riesj@uni-trier.de; 2 Dienstleistungszentrum Ländlicher Raum Mosel, 54770 Bernkastel-Kues, Germany; 3 Soil Erosion and Degradation Research Group, Department of Geography, University of Valencia, 46010 Valencia, Spain; 4 Zentrum für Geoinformationswesen der Bundeswehr, 53879 Euskirchen, Germany; stefan2wirtz@bundeswehr.org

**Keywords:** linear erosion, geo-sensors, TEPHOS, environmental monitoring, runoff simulation, Structure from Motion (SfM)

## Abstract

The Terrestrial Photogrammetry Scanner (TEPHOS) offers the possibility to precisely monitor linear erosion features using the Structure from Motion (SfM) technique. This is a static, multi-camera array and dynamically moves the digital videoframe camera designed to obtain 3-D models of rills before and after the runoff experiments. The main goals were to (1) obtain better insight into the rills; (2) reduce the technical gaps generated during the runoff experiments using only one camera; (3) enable the visual location of eroded, transported and accumulated material. In this study, we obtained a mean error for all pictures reaching up to 0.00433 pixels and every single one of them was under 0.15 pixel. So, we obtained an error of about 1/10th of the maximum possible resolution. A conservative value for the overall accuracy was one pixel, which means that, in our case, the accuracy was 0.0625 mm. The point density, in our example, reached 29,484,888 pts/m^2^. It became possible to get a glimpse of the hotspots of sidewall failure and rill-bed incision. We conclude that the combination of both approaches—rill experiment and 3D models—will make easy under laboratory conditions to describe the soil erosion processes accurately in a mathematical–physical way.

## 1. Introduction

Soil loss is one of the most important causes of land degradation processes, which generates a loss of soil fertility and biodiversity in both natural and anthropogenic soils [1,2,3,4]. An excess of surface runoff due to the presence of bare soils and extreme rainfall events are two of the main reasons for global soil loss in arable, forested, burned or urban lands [5,6]. Moreover, paying attention to the imminent climate change impacts, overpopulation and water scarcity, soil erosion is leading to environmental degradation and, subsequently, to an undesirable loss of productivity [7,8]. In this way, several authors agree that one of the most important volumes of sediment detachment is generated as a consequence of rill and gully erosion [9,10,11].

Linear erosion, in the forms of (ephemeral) gullies and rills, is considered as one of the most important issues concerning soil surface, especially because of the possible dramatic consequences such as landslides, floods or landscape fragmentation [12,13,14]. Rills and gully erosion can generate more or less persisting forms, which can evolve into gullies and deteriorate any productive soil surface [15,16]. However, due to the intra-plot variability of pedological properties, parent material, land-use practices and climate conditions, the development of these linear erosion features are mostly unclear and must be further studied [17,18,19]. Several authors have remarked that studies focusing on rill and gully erosion should pay attention to the connectivity and des-connectivity processes from the hillslope to the watershed scales because several driving factors play a key role in runoff and sediment yield activation [20,21].

In recent decades, numerous studies on rill and gully erosion have been conducted using models, connectivity indexes, morphological measures, light detection and ranging (LiDAR) data or remote sensing techniques [22,23]. Thanks to these ever-increasing computing capacities, it has even become possible to model soil erosion with a higher spatial resolution in close connection with climate prognosis models [24,25]. Regarding this fact, important advances have also been addressed thanks to the laboratory analysis. Under laboratory conditions, external factors, such as wind, soil moisture, vegetation, and rock fragment cover or even the inclination, can be controlled, enhancing the final interpretation of the results [26,27]. However, the reality can be altered and an increase in the difficulty to extrapolate the data to the natural conditions can be found. Therefore, experimental designs under field conditions may enhance the accuracy of the data and their reproducibility.

To measure rill and gully erosion under field conditions, several examples using runoff simulators can be found in the recent literature. For example, Kavian et al. [28] used a runoff simulator to test the efficiency of buffer strips to reduce soil erosion in abandoned hillslopes in Iran. In Spanish conventional vineyards, García-Díaz et al. [29] also conducted runoff experiments aimed to investigate the water and nutrient losses, specifically, nitrogen, using different cover crops in close plots. Also, the standardised runoff simulation experiment developed by Trier University has been widely applied in Mediterranean areas using 1000 L of water to assess sediment yield, hydraulic parameters and morphological changes [30]. As this method was developed to be standardised, its reproducibility conforms an advantage like other field methods such as the small portable rainfall simulator [31,32], single-ring infiltrometer [33,34] or Guelph permeameter [35,36]. Badlands, almond trees or agri-spillways in vineyards are some examples where this runoff simulation method has been successfully applied [37,38]. However, currently, information about the exact volume of soil loss and morphological changes at the micro-pedon scale, which could help to assess which driving factor (e.g., rock fragments, biota or inclination) is acting more actively, is scarce. This interpretation cannot be conducted using general rates such as sediment concentration or runoff coefficient, measurements pre- and post-experiments using meter-tapes or general pictures. Therefore, the use of photogrammetry techniques would be the best solution to fill this gap.

Structure from Motion (SfM) as an upcoming subtype of photogrammetry, and it is largely used in different fields of science such as surveying, architecture, archaeology, geography and others [39]. In land management and soil sciences, SfM is also widely applied to detect and quantify changes caused by certain land degradation processes and their distribution in landscapes or integration into soil mapping procedures [40,41]. The necessary pictures for this type of change detection are usually taken with the help of platforms such as drones, airplanes, helicopters and multicopters [42,43]. However, the combined use of runoff experiments under field conditions and SfM photogrammetry is insufficient. Some developments on this topic have been published using new software, mobile phones or drones [19,44,45] but not in a combination of scale, active rills, or gullies and all this combined with extreme runoff conditions.

Thus, the goals of this methodological approach were to (1) obtain better insight into the rill erosion processes; (2) reduce the technical gaps generated during the runoff experiments using only one camera; (3) enable the visual location of eroded, transported and accumulated material. To achieve this goal, we developed the Terrestrial Photogrammetry Scanner (TEPHOS). To demonstrate the effectiveness of this device, we show a merely traditional photogrammetry procedure by using two cameras in a stereo array. After that, we improved TEPHOS by using five cameras. We hypothesized that the resolution and the quality of the models during runoff experiments could be highly improved.

## 2. Materials and Methods

### 2.1. The Runoff Simulation Experiment Procedure

**Figure 1:**
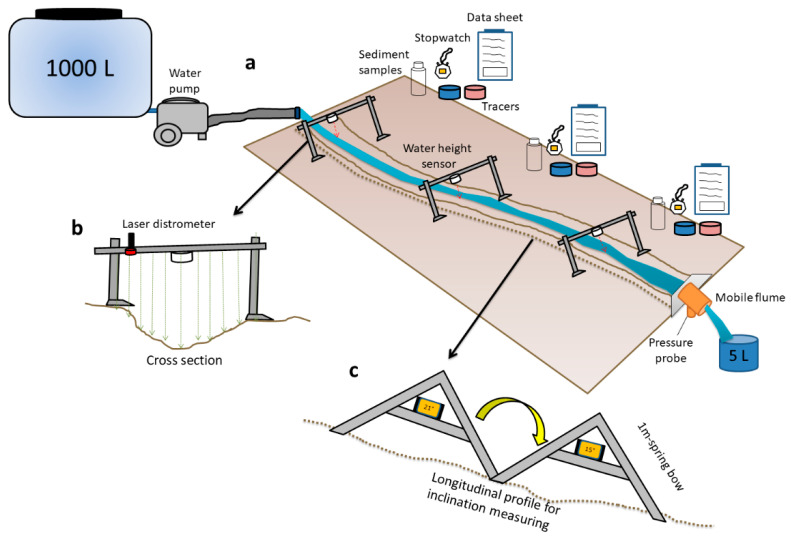
Runoff experiment procedures: (**a**) rill experiment with the different measurement points; (**b**) cross-section; (**c**) longitudinal profile measurements.

The runoff simulator developed by Wirtz et al. [27] was designed by Trier University to compare active soil erosion processes and hydraulic parameters of rills and gullies from different territories under controlled and known extreme conditions. In Figure 1, a presentation of the whole experiment is presented.

The runoff simulation consists of two runs under (1) field capacity conditions (run a) and (2) saturated soil conditions (run b), more or less after 15 min. In Figure 1a, we can observe that a constant water discharge (≈250 L min^−1^) was pumped over 4–6 min reaching a total water inflow of approximately 1000 L. We tried to avoid the mobilisation of material at the initial inflow with the use of an attenuator [46]. We disposed of three measuring points (MP1, MP2 and MP3), and the water height was measured using a sensor. After the first run (a), the cross-section was measured in order to observe micro-topographical changes using a laser rangefinder (Figure 1b). During the experiments, a total of four water samples were collected, firstly, when the waterfront reached the MP and after 30, 90 and 150 s later. Then, in the laboratory, the sediments suspended in the water (sediment concentration) were estimated filtrating the water samples.

Time was controlled using a digital stopwatch in each measuring point. Moreover, three people equipped with datasheets had to monitor the water speed using soluble red (E124) and blue (E13) tracers. Three velocity curves were registered and modifications in flow dynamics were evaluated. As the obtained flow velocities were not equal to the flow velocities at sampling time, we hypothesized two possibilities: a linear increase or decrease among the three measured flow velocities. This was necessary to estimate the velocities among those points to obtain the velocities for the sampling time [46]. At the end of the rill or gully, a mobile flume and pressure probe (Ecotech DL/n, V2.35) measures removed water discharge at different time intervals. We also calibrated the discharge curve, recording the runoff at the outflow at regular intervals. This was necessary to measure the constant water discharge with enough temporal resolution during the runoff simulation. After each experiment, the local slope was measured using a spring bow and a digital clinometer. It must be considered that the local inclination data provide only the mean values for 1 m; therefore, micro-morphological changes cannot be accurately detected, but their position and height were recorded in a sketch.

**Figure 2:**
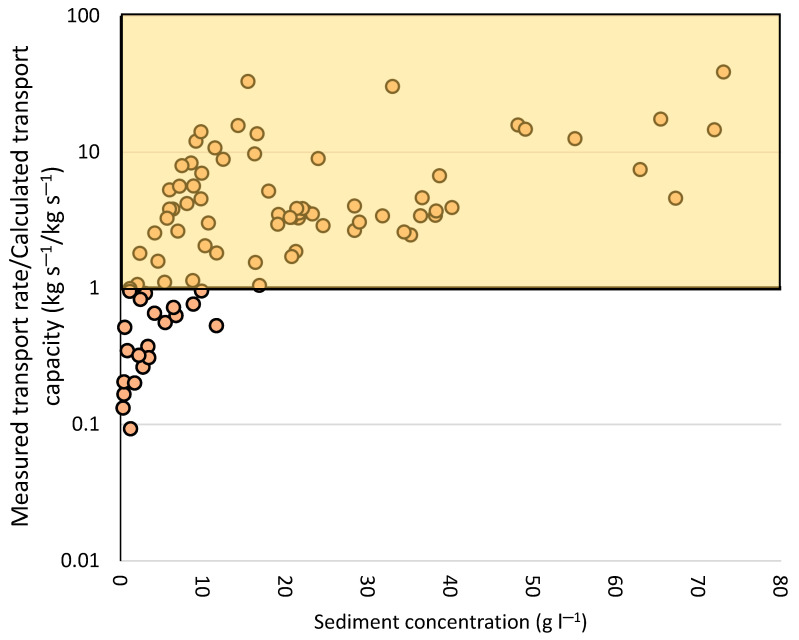
Predicted sediment transport capacity in 67 runoff simulations (modified by Wirtz et al. [46]). Points in orange above 1 show the measured values that exceed the predicted maximum transport capacity. Values on the *y*-axis > 1: the rate is higher than the capacity).

After performing the runoff simulation, morphological changes (e.g., inclination and cross-sections), hydraulic parameters (i.e., water level changes, flow velocity, water discharge) and soil erosion results (i.e., suspended sediment concentration) could be measured directly. Moreover, after using the Water Erosion Prediction Project (WEPP) software, transport rate and transport capacity can be modelled. Wirtz et al. [46] calculated these parameters mentioned above from 67 runoff experiments. Comparing the data already collected in the experiments with the values predicted by WEPP, these authors noted that the real transport rate exceeded the predicted transport capacity, which, in theory, is not possible (Figure 2).

We consider this fact remarkable and confirm that micro-topographical changes may be primarily responsible for this transgression in the predicted values. Thus, the key question would be: which parameter was not surveyed related to the non-measured values? Except for the three cross-sectional measurements (Figure 1b) where each measurement station was installed, little is known for instance about the changes of the internal micro-topography along the whole rill. Therefore, we affirm that it would be necessary to know more about these morphological changes in order to quantify the real transport capacity during the water discharge. We hypothesized that the results of the measurements could be influenced by undetected sidewall failures, plunge-pool dynamics or direct depletion.

In summary, we tried to demonstrate that the most important disadvantage of the runoff simulations, such as the rill experiment developed by Trier University, was the lack of information about the micro-topographical changes between the cross-section and the measuring points because of the absence of non-intermittent surveying capabilities. To close the data gap between the three measuring points, a continuous tool for topographic data acquisition had to be contrived. We proposed the Structure from Motion photogrammetry to achieve this goal and, in addition, the development of a device optimised for terrestrial-based photogrammetry.

### 2.2. Structure from Motion (SfM) Adapted to the Runoff Simulations: the TEPHOS

Structure from Motion allows assessing any surface by collecting a specific number of photos from different points [47,48], resulting in a continuous point cloud of the surveyed terrain. If there are point clouds computed before and after performing the experiments, a difference image could show the outcomes of the processes altering the micro-topography inside the surveyed plot. In the abovementioned runoff experiment, where the consequences of torrential rain on agricultural surfaces are simulated, the changes caused by the experiment can then be examined in detail.

The overall resolution of the 3D model must be orientated to the size of the smallest targets of interest. The limits of the target’s sizes are considered to be from sand grains via soil conglomerates to little stones with dimensions from millimetres to centimetres. We configured the device according to these aims. The accuracy of the 3D model is also a result of the condition of the photoset [49,50]. Therefore, an affordable device capable of surveying the erosion rills under observation was designed: the TEPHOS.

#### 2.2.1. Study Area

We proposed a study case based on a hand-made rill in a forestry area in Luxembourg of approximately 10–20 m in length and 0.2–0.3 m in width and depth. The research area is located in Central Europe, in the Grand Duchy of Luxembourg in the catchment area of the Our. This has a catchment area of approximately 670 km^2^ and is 78 km long. The Our River has 29 tributaries, one of them is the Feierbech (in German: Feuerbach) where this experiment was conducted close to the village of Kalborn (50° 06′ N; 06° 07′ E). It has a total contributing area of about 1.19 km^2^ and a variety of land uses such as arable land, grassland, forest, and settlements. The parent material consists of slates and quartzite from the lower Devonian. This area is part of the Ardennes, which belong to the Rhenish Slate Mountains. It was created at the time of the Variscan orogeny. The wide and open plateaus are located at approximately 450 m a.s.l.

#### 2.2.2. TEPHOS

**Figure 3:**
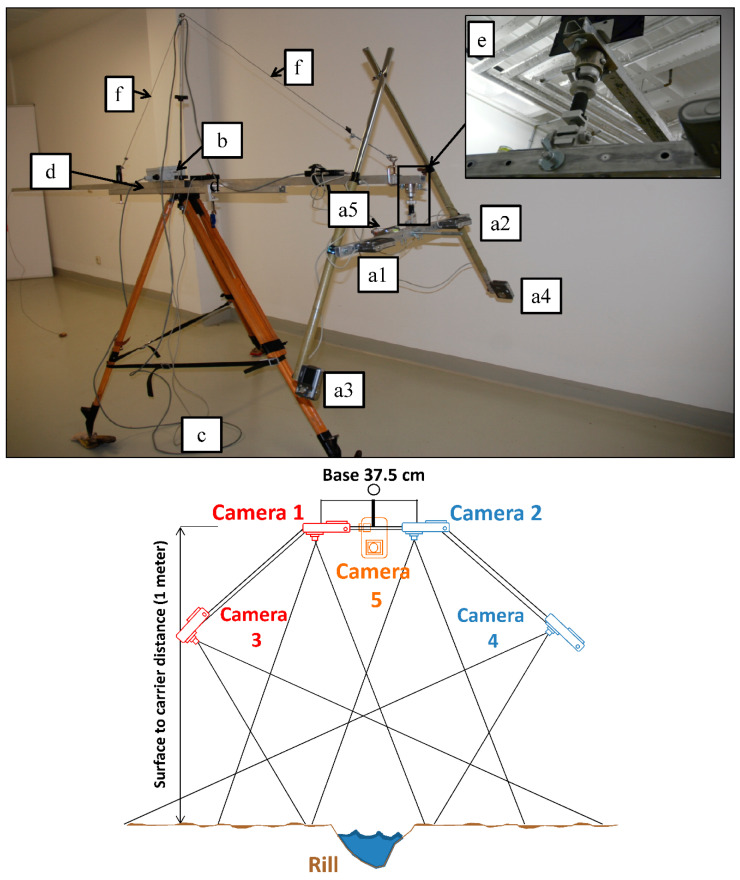
TEPHOS (Terrestrial Photogrammetric Scanner). From image (**a1**) to (**a5**): cameras; (**b**): battery; (**c**): tripod; (**d**): telescopic arm; (**e**): spherical joint; (**f**): guy wire.

In Figure 3, the TEPHOS is presented here as our possible solution to assess the consequences of active soil erosion processes during the runoff simulations. This device began development in 2011 and the last tests under field conditions were conducted in 2018 [51].

Five synchronised Nikon L2 consumer digital cameras (Figure 3a1–a5) were considered to obtain both perpendicular and oblique views on the rill’s sidewalls. They have 3× Zoom-Nikkor lenses with 6.3–19.2 mm (≈38–116 mm). An f/3.2–5.3 and lenses packed into five elements and different groups were connected to be able to obtain approximately 6.0 million effective pixels. In addition, a sensor format of 1/2.5 inch and size of about 24.7 mm^2^ (5.76 mm × 4.29 mm) were used. Finally, a near pixel pitch of 2.05 microns was included. All of them can work at the same time using a tethered remote-control unit. This could also serve as a constant power fed by a 12 V/48 Ah car battery (Figure 3b). The cameras were installed on an aluminium rail symmetrically to adjust the TEPHOS to diverse requirements (Figure 3c). The rail was incorporated to a telescopic arm of 3.5 m by a spherical joint to get a gimballed arrangement (Figure 3d). The link between both structures was conducted by clamping (Figure 3e,f).

At the beginning of the evolutionary process, we used only two Nikon L2 cameras placed in a stereo arrangement with an altitude above ground of 1.00 m on and a base distance of 37.5 cm. We adjusted the camera’s zoom lenses to a wide-angle mode with a focal distance of 38 mm [52]. The main idea was that each picture overlapped by approximately 80% in a principal direction and approximately 60% in the orthogonal direction. Due to the undesirably of data gaps and model furling, we decided to add two side-looking cameras (Figure 3a3,a4) and a 90° offset camera (Figure 3a5).

As a next step, the requirements for SfM-serviceable photos were designed considering an ideal crop and suitable scale. We paid attention to ensure the focal length, correct exposure, high contrast, sufficient sharpness and enclosing depth of field (with all questions of shutter speed and aperture). Minimal blur and hemispherical exposure points were also considered. We observed that as the main disadvantage, the demands for storage and transport space could be considered as unfavourable.

To improve the accuracy acquired by the lowest possible shutter speeds and to reduce the post-processing time using systematisation, we use the fixed array of five Nikon L2 consumer cameras. As a result of fixing the cameras and using a constant array-feed of ten centimetres, we were able to eliminate data gaps thanks to the crisp sharp pictures and the sufficient overlap in all directions. A schematic depiction of the photo set matrix is presented in Table 1.

Other advantages of using that configuration are the same lighting conditions for at least five synchronously taken pictures. If needed, the TEPHOS can provide better visibility of sidewalls as a result of the 45° side-hanging cameras. This was due to the elimination of errors implanted through the use of different camera models. In addition, there was no furling of long, narrow objects, for example, straight rills, which meant that the nautilus or dome effect (the furling of long, narrow objects) was reduced or avoided by the addition of the 90° camera (Figure 3a5). Last on the list of advantages was the chance for the operator to get five photos at once without shifting positions. On the other hand, this configuration meant increased costs for five identical cameras and precarious intrusions in all of the camera bodies for connecting the electronic synchronisation and power supply.

#### 2.2.3. Image Treatments

In order to compare the resulting post-experiment surface (t_1_) to the pre-experiment surface of t_0_ (initial time), the point cloud (t_1_) was converted into a mesh by computing a very dense wireframe model. The operator was then enabled to subtract the mesh of t_1_ from the initial surface (t_0_). The resulting difference image will directly show the resulting modifications in the linear feature. To achieve these image treatments, the AGISOFT PhotoScan software (version 1.3.5., St. Petersburg, Russia) and CloudCompare (Telecom ParisTech, version 2.1., Paris, France) were used.

First, AGISOFT PhotoScan computes the 3D models based on the photos made in the above-mentioned way. This means that all five photo sets were loaded onto a solid-state drive for more computing velocity. To proceed with the images’ treatments, we performed: (1) adjusting preferences settings; (2) loading photos from all five cameras; (3) aligning all photos, optimizing the camera alignment and building the sparse cloud; (4) building a dense point cloud, allowing, finally, for PhotoScan to build the mesh.

**Figure 4:**
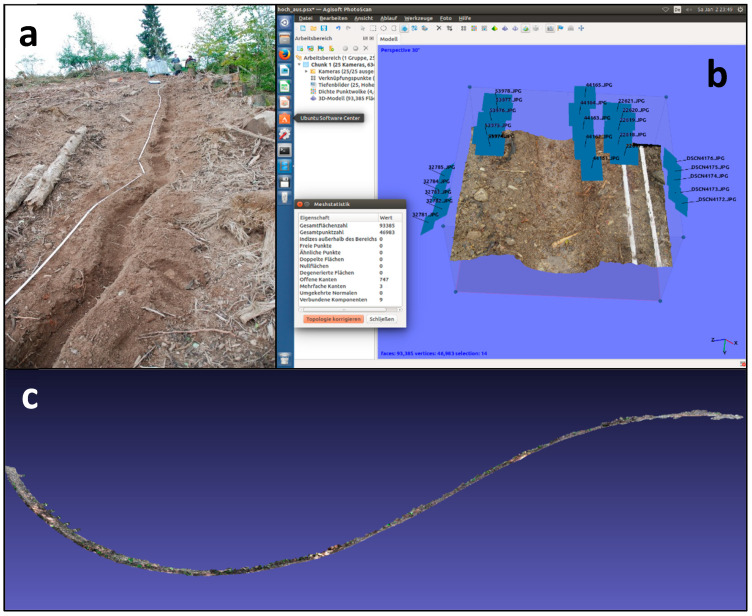
Runoff simulation and image treatments: (**a**) runoff simulation; (**b**) image treatments in AgiSoft software; (**c**) “nautilus” effect, furling a rill of 20 m long.

We preferred this software to other ones because of its ease of use and its accuracy. Another advantage is processing security, especially while handling big photosets. For a usual erosion linear feature survey of 20 m length (Figure 4a), we process 1000–1500 pictures, which takes around 3–4 days in total. Also, this software is able to perform photogrammetric processing of digital images and generate 3D spatial data by calculating the corresponding propagation of uncertainty (Figure 4b). In our case, it was used to perform the panorama stitching, triangulation and creation of the point clouds and meshes. The adjustment of the controlling elements was always aimed at the highest possible accuracy.

After image processing, the next step was the comparison of the two-point clouds with the software CloudCompare. CloudCompare is a processing software prepared for the investigation of 3D point clouds and triangular meshes. It was originally designed to perform comparisons between two dense 3D points clouds acquired with SfM or LiDAR techniques or among point clouds and triangular meshes. It relies on a specific octree structure dedicated to this task. We used the comparison module to compute the different models of the pre-experimental model (t_0_) and the post-experimental one (t_1_). The difference model, induced by subtraction of model (t_1_) minus model (t_0_), will then lead us to the points of interest in the rill area, like collapsed banks, segments with a lot of incisions, steps and others. The workflow was easy and quickly conducted, as both point clouds and meshes have to be loaded and, then, the subtraction can be performed.

### 2.3. Accuracy Assessment

To estimate the accuracy obtained from the processed images, two different methods were applied. Firstly, it relies on PhotoScan. We printed out the error per pixel obtained in the image, using the root mean square re-projection error over all the feature points detected. Moreover, by using a scale bar on the photos, there was the possibility to compare the real length of the distances on the ruler under field conditions and with the corresponding lengths of the modelled ruler. The scale bar section would provide us with the information on the error difference between the input (source) scale bar length and the measured distance between two cameras or markers. This also represents the start and endpoints of the scale bar.

The second method is related to the calculations of the point density on the model, which can be calculated as follows (Equation (1)) considering previous research [53,54]:

(1) $$\mathrm{Number}\;\mathrm{of}\;\mathrm{points}\;\mathrm{inside}\;\mathrm{the}\;\mathrm{area}\;(\mathrm{length}\;\times \;\mathrm{width})\;=\;\frac{\mathrm{Number}\;\mathrm{of}\;\mathrm{points}}{\mathrm{Area}\;\left(m2\right)}$$

In addition to this, we also looked at the picture density for each point’s calculation.

## 3. Results and Discussion

### 3.1. Results Obtained Using Only a Stereo Device

**Figure 5:**
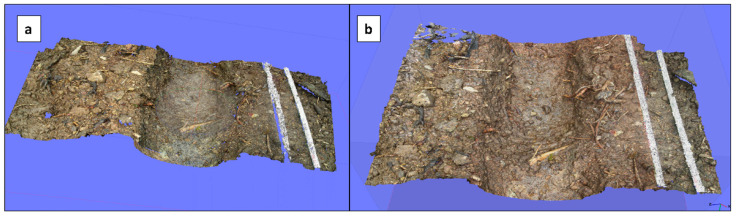
Comparison of meshes by the stereo device (**a**) and the Quadro device (**b**).

During the design period, the first experiments under laboratory conditions were conducted using only two Nikon L2 cameras in the near-nadir arrangement. The results showed that it is possible to compute 3D models in this way, but data gaps, especially under overhanging obstacles and vertical objects, were registered as other authors have indicated [55,56]. These data gaps are the outcome of a missing stereo-coverage. In Figure 5a, we observe that data gaps can be found especially in those areas, where the direct view from the near-nadir cameras is hindered by obstacles. Thus, especially here, a side-looking capability from both sides would have been favourable to ensure sufficient overlapping, which coincides with Cullen et al. [57] while measuring onshore erosion platforms. As a result of these observations, we intended to mount additional cameras as Zimmer et al. [58] did to monitor soil erosion due to the footprints in Tanzania.

### 3.2. The Implementation of Two Extra Cameras: The Quadro Device

The mounting of two additional outriggers and two cameras with versatile angles (Quadro device) provided a better model quality, especially in terms of three-dimensionality, coherence and accuracy. According to Mallison and Wigs [59], it is recommendable to use the same structure in order to reduce possible scaling errors. The detection of changes inside the walls and on the ground of deeply incised rills was of special interest because processes like sidewall failure and incision are likely to occur here [60,61]. In Figure 5b, the comparison of the stereo model and the Quadro model showed the improvements clearly. The stereo model on the left side was slightly bigger by real size than the Quadro model on the right part, but the total amount of points (3,921,453 points) was far below the 4,522,077 points of the piece obtained from the Quadro device. The stereo model showed some data gaps in the area of the folding metric yardstick, which were eliminated by the use of the additional side-looking cameras.

In this step, we tried to get eliminate the nautilus effect, which meant the nautilus-like furling of long and narrow objects in the models. The first solution approach dealt with changing the software, but to cycle through different programmes did not alter the results and the models kept furling. The next possibility was a camera with an offset of 90°, as all the furling seemed to arise by having the existing cameras, their lenses and the lenses’ errors orientated in the same direction. Finally, the decision to use the offset-90° camera proved useful as the furling stopped.

In order to obtain insights into how the accuracy of this last evolution would be, we modelled the dense point cloud of a rill segment of approximately 60 cm × 60 cm. This segment was computed by using 5 × 3 pictures, a scale bar and the highest possible accuracy settings. After the first visual verification, errors had to be predicted. To calculate/predict the error, the prediction/calculation tool of AGISOFT PhotoScan was used. The data delivered for one example can be observed in Table 2 using 30 markers that were installed all over a 20 m long area (<1 cm).

In this example, we can observe the mean error of all pictures reached to 0.00433 pixels (ground area per pixel ± 0.005347 m × 0.005347), and every single one of them was under 0.15 pixel. So, we obtained an error of about 1/10th of the maximum possible resolution. Although the results can be considered acceptable, it seems that the operator was a possible limitation. In PhotoScan software, the scale bar has to be placed in the 3D model by the operator manually. This procedure can only be done with a maximum accuracy of one pixel. Concerning this, a conservative value for the overall accuracy is one pixel, which means that, in our case, the accuracy was 0.0625 mm.

**Figure 6:**
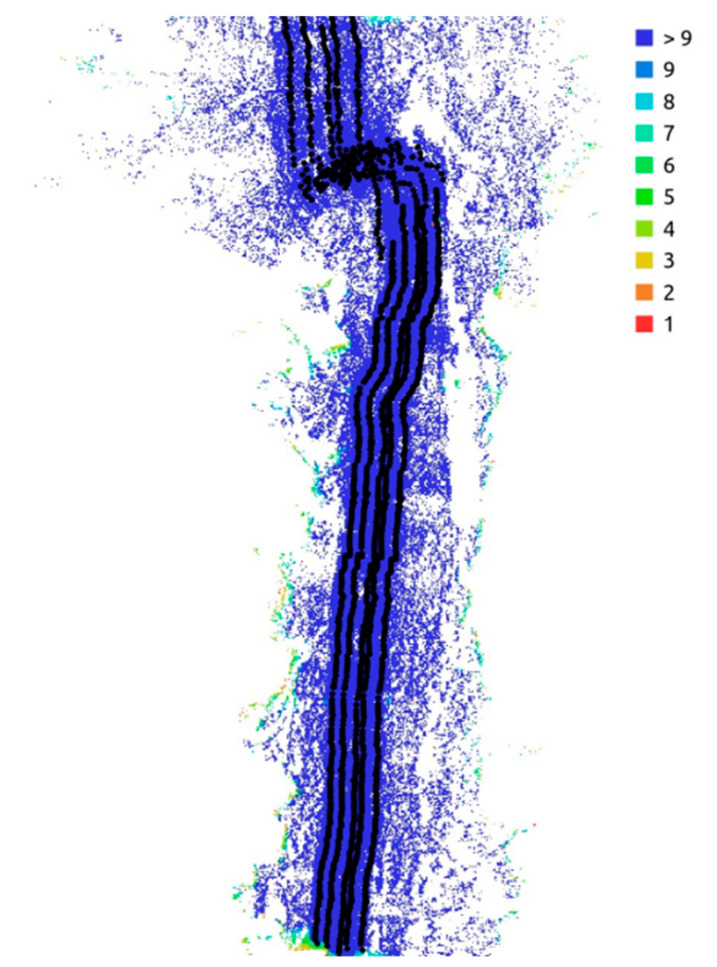
Ground coverage and the number of images per point.

The second value of interest—the point density—in our example reached 29.484.888 pts/m^2^. As this value is a mean (points/area), it does not fit the real situation, because the reality is seldom flat, and so the distribution of the point on the surface is not homogeneous. For example, on a slope, the point density is higher than in a flat area, so point density may only be a supplementary tool to access a models’ quality. Figure 6 shows how many images can be simultaneously be used to compute one single point in the point cloud. Nearly 100% of the area of interest is colour-coded in dark blue, which states that more than nine pictures from different directions or angles are available to compute all the points in the rill and its surrounding area. Taking into account the way SfM works, we can assume that this high number of images per point could lead to good accuracy and certain processing confidence.

**Figure 7:**
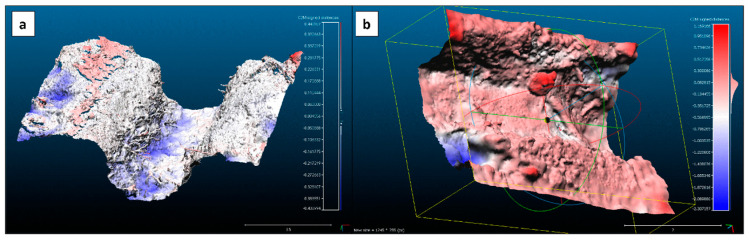
Final model using TEPHOS. (**a**) Model used to highlight the effect of roots and soil structure; (**b**) model used to highlight the effect of the embedded rocks.

Using the scale bar in PhotoScan, we can read off an error of about 0.000071 m. This value again shows that the accuracy obtained by the fixed array setup is more than sufficient for our task. In the following example, the capabilities of the experiment—TEPHOS + software combination—are presented. The typical identification of geomorphological processes in the micro-topography is presented in Figure 7, which shows the division model of two meshes, t_1_–t_0_, which suggests a lowering of the rill-bed. The blue colour-code indicates erosional digging of approximately 1 cm in-depth, which may be caused by turbulently flowing water. The turbulence seems to be caused by small obstacles, in this case, roots and a step in the rill’s bed; this was also observed under laboratory conditions modelling the flow resistance of rills [62,63].

The sum of the model t_1_–model t_0_ shows that there was erosion at the bottom of the rill segment. The erosion from 15 to 20 cm (blue colour) may be caused by incision and depletion. Red coloured areas represent the occurrence of accumulation. All the areas colour-coded in white, state that there was no change, which is a positive sign in terms of functionality of the setup.

Two relevant areas can be highlighted. In the lower-left corner, we can observe a deep bluish colour-coded hollow pattern. It seems to be a phenomenon caused by flowing water [64,65]. In the middle of the section, we find a pebble-like pattern of accumulation of about 1.1 cm of diameter. The detection of rock fragment cover and classification into different groups depending on the position along the eroding rill is key to understanding water infiltration, soil particle mobilisation, and flow velocity [66,67]. In this example, most of the rill section shows no accumulation (light pink) and minimal depletion is detected. The overall sparse alteration in unaffected parts shows the homogeneity of both models.

**Figure 8:**
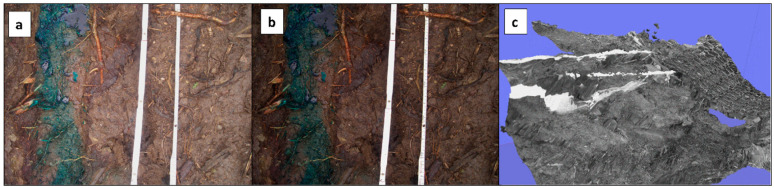
Application of contrast to enhance details. (**a**) and (**b**) contrast enhancement; (**c**): data results.

While optimising platform, device, and procedure, we found some spillover. Most of the following is resulting from the need to shorten the computing time. In many areas of application, remote sensing techniques of image enhancement, such as filtering, data combination, contrast enhancement, and others, are widely used [68]. In our case, we applied contrast enhancing on details of the folding yardsticks. This improvement measure can be observed in Figure 8a,b, which shows parts, often overexposed due to the darker background evoked by the saturated soil conditions.

In order to reduce the computation time, some procedures with monochromatic data were carried out. The outcome of this experiment is shown in Figure 8c, which decreased drastically the computation time. On the other hand, the number of feature points was also drastically reduced (colour: 4 mio. feature pts. to black and white: 103.000 feature points), which leads to weaknesses in the edge areas of the resulting models that are visible in Figure 8c. We assumed that the reduction of distinguishable features by excluding the colour information leads to an unfavourable reduction of tie-points.

### 3.3. Challenges and Future Procedures

A synchronised, multi-camera array combined with a runoff experiment was used to assess active processes in rills caused by a runoff experiment. The results showed that it is possible to detect the remnants of surface runoff. For the operator, it was possible to analyse the whole 20 m rill of the runoff experiment with special respect to processes such as sidewall failure, plunge pool dynamics, incision and others. The SfM photogrammetry is usually more often used under laboratory conditions and on a larger scale in geomorphology [69,70], but we demonstrated that it could reasonably be used for measurements at the pedon scale and, especially, for the detection of leftovers of active soil erosion processes. Unfortunately, structure from Motion photogrammetry does not deliver an exact explanatory formula for the processes that alter the micro-topography.

We agree with Zimmer et al. [58] that with a carefully planned photo-survey the microtopography measuring device with consumer cameras could commend itself as a low-cost substitute for a laser scanner in erosion-orientated close-range-photogrammetry. With its high accuracy in mind, it may, for example, be of help to compute soil-roughness [71,72]. On the other hand, in terms of costs, it is useful to know that the camera array is not the limiting factor, but the computer system, which will oversee calculating the 3D model [51]. As some of the calculating is done on the graphics card, a grouped workstation is favourable, otherwise, models are not processed as one continuous pattern.

As a spillover, we found some interesting facts, such as that the number of pixels of the surveying camera is of minor importance than the quality of the employed optics, platforms and planning of the “flight” [73]. This result stands in some contrast to the idea of processing some rills by smartphone and cloud [45,74], as the optics of smartphone cameras usually are operating with tiny sensors of enormous pixel numbers with an unfavourable signal/noise-ratio and heavy distortion, which might lead to unsatisfactory results [75]. For micro-topographical concerns, it may be advantageous to sneak a peek at industry photogrammetry where results in sub-millimetre accuracy are archived by using mounted multi-camera arrays [76].

## 4. Conclusions

In this article, we showed how to survey the outcome of a runoff experiment made in order to obtain a clearer insight into rill erosion processes. We use a fixed array of five similar cameras that were synchronised and power supplied to get photosets of the accuracy of approximately 1 mm^2^ per pixel. Five cameras, including stereo near-nadir cameras, side-hanging cameras on outriggers and a single 90° offset camera allow the modelling and later on the analysing of a 20 m long segment of an erosion rill flushed during the experiment. The subtraction model allowed the detection of changes in a way that is impossible while in the field because angles and optical magnification could be altered on demand. In the near future, it will be possible with little effort to strike the balance of erosion and accumulation of segments or points of interest inside the rill and do a calculation in mg/m^2^. As a last benefit of modelling continuously in 3D, the problems of interpolating the data between the cross-sections and finding the correct upper border of the rill can be dropped. As quite a disadvantage, we have to take into account the vast computational time, which depends on the machine used, but as workstations are becoming more capable and less expensive, this disadvantage will shrink.

## Figures and Tables

**Table 1:**
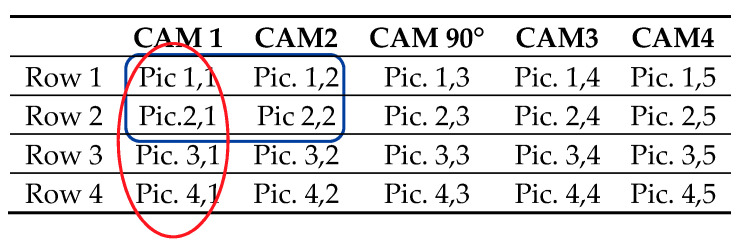
Identification of stereo pairs by positional matrix. The ovals mark the overlapping pairs in their respective direction.

**Table 2:** Error (pix): root mean square re-projection error calculated over all feature points detected on the photo.

Markers	Error X (m)	Error Y (m)	Error Z (m)	Accuracy (m)	Error (m)	Projections	Error in px
point 1	−0.008	0.001	−0.004	0.005	0.009	20	0.004
point 2	−0.008	0.001	−0.004	0.005	0.009	20	0.004
point 3	0.006	−0.0002	0.002	0.005	0.006	16	0.003
point 4	0.006	−0.0003	0.002	0.005	0.006	11	0.004
point 5	0.004	−0.001	0.001	0.005	0.005	20	0.003
point 6	0.004	−0.001	0.001	0.005	0.005	22	0.003
point 7	0.004	−0.001	0.001	0.005	0.004	27	0.004
point 8	0.004	−0.001	0.001	0.005	0.004	29	0.004
point 9	0.004	−0.001	0.001	0.005	0.004	23	0.003
point 10	0.004	−0.001	0.001	0.005	0.004	24	0.003
point 11	0.004	−0.001	0.0001	0.005	0.004	15	0.003
point 12	0.004	−0.001	0.001	0.005	0.004	7	0.004
point 13	0.003	−0.001	0.001	0.005	0.003	17	0.003
point 14	0.003	−0.001	0.001	0.005	0.003	17	0.003
point 15	−0.001	0.0001	−0.001	0.005	0.001	39	0.004
point 16	−0.001	0.0002	−0.0001	0.005	0.001	40	0.003
point 17	−0.009	0.001	−0.004	0.005	0.010	17	0.004
point 18	−0.009	0.001	−0.004	0.005	0.010	18	0.004
point 19	−0.005	0.001	−0.003	0.005	0.006	15	0.003
point 20	−0.005	0.001	−0.003	0.005	0.006	15	0.003
point 21	0.007	0.001	0.002	0.005	0.008	5	0.021
point 22	0.008	0.001	0.002	0.005	0.007	4	0.024
point 23	−0.003	0.0002	−0.001	0.005	0.003	10	0.004
point 24	−0.003	0.0003	−0.001	0.005	0.003	12	0.004
point 25	−0.004	0.0005	−0.002	0.005	0.005	16	0.004
point 26	−0.004	0.0005	−0.002	0.005	0.005	17	0.003
point 27	−0.001	0.0002	−0.0005	0.005	0.0008	34	0.003
point 28	−0.001	0.0002	−0.0005	0.005	0.0008	36	0.003
point 29	0.001	6.727	3.0655	0.005	0.0006	26	0.004
point 30	0.001	6.66	3.9304	0.005	0.0007	27	0.003
Total error	0.005	0.006	0.002		0.005		0.004
